# Thermal Degradation of Face Mask Waste through Pyrolysis at Different Temperatures for Value-Added Products

**DOI:** 10.1038/s41597-025-05269-1

**Published:** 2025-05-28

**Authors:** Nikola Cajova Kantova, Michal Holubcik, Robert Cibula, Katerina Benova, Jachym Muck, Jan Najser

**Affiliations:** 1 Research Centre, University of Žilina, Univerzitna 8215/1, 010 26 Zilina, Slovakia; 2 Department of Power Engineering, Faculty of Mechanical Engineering, University of Žilina, Univerzitna 8215/1, 010 26 Zilina, Slovakia; 3 ENET Centre, CEET, VSB-Technical University of Ostrava, 17. listopadu 15/2127, 708 00 Ostrava, Czech Republic

**Keywords:** Chemical engineering, Energy science and technology

## Abstract

The widespread use of protective face masks to prevent the transmission of viral diseases has led to a significant increase in plastic waste. This study investigates the conversion of disposable face masks into valuable solid, oil, and gas products through pyrolysis. Two types of disposable masks were used as input materials: FFP2 face masks and ordinary face masks. The pyrolysis reactor was heated to various target temperatures (350, 400, 450, 500, and 550 °C). Particular attention was given to a detailed analysis of the physicochemical properties of the raw materials and the characterization of the pyrolysis products at different temperatures. The measured data provide a basis for analyzing the pyrolysis process of face masks and respirators under varying thermal conditions. Identifying the relationships between input materials, the pyrolysis process, and the properties of the resulting products could enable the production of targeted products with high added value.

## Background & Summary

Despite a globally observable increase in hospital waste, the COVID-19 pandemic has markedly accentuated this trend. This development has been reflected in a growing number of scientific publications focusing on the issue of medical waste and its impact on the environment and public health^1–3^. A substantial proportion of hospital waste is comprised of single-use protective equipment, particularly face masks and FFP2 respirators^2,4^, which were widely used as preventive measures not only within healthcare facilities but also among the general population. The increased consumption of this equipment led to a rise in the volume of infectious waste, creating the need for efficient, safe, and controlled disposal methods to prevent further spread of viral contaminants^5^.

Due to the presence of viral contaminants, it is essential to ensure safe and controlled disposal methods to minimize the risk of further infection transmission. Conventional approaches to medical waste management (such as incineration or landfilling) have several disadvantages, including permanent occupational hazards, environmental pollution, and the generation of harmful emissions^6^. To address this issue, the implementation of controlled collection systems, such as strategically placed collection containers, is recommended. At the same time, strategies for material recovery and energy valorization of this waste should be developed^7^. Suitable technologies for such purposes include mechanical and thermochemical processes.

Among thermochemical treatment options, incineration, gasification, and pyrolysis are well-established. Incineration primarily yields heat, whereas gasification produces syngas, which is valued for its energy content. Several studies^8,9^ have highlighted the feasibility of converting face masks into hydrogen-rich syngas via gasification using integrated processing systems. In the case of pyrolysis, the products are a mixture of liquid, gaseous, and solid fractions – with the dominant product depending on the process parameters.

Among thermochemical processes, pyrolysis stands out as a suitable technology for treating this type of waste. It enables the transformation of face masks and respirators –shown in studies^1,2^ to contain large amounts of plastic – into products that can be further utilized. These products have potential applications primarily in the chemical industry, but also in the energy sector. This thermochemical process takes place in an inert atmosphere (i.e., without access to oxygen), which results in minimal emissions and eliminates the need for extensive pre-treatment (except for appropriate particle size adjustment), offering a safe and environmentally responsible solution for processing contaminated medical waste^10^.

Although the pyrolysis process primarily yields products suitable for chemical use (e.g., for fuel or plastic production) rather than direct energy recovery^11^, in their study^12^, Sari *et al*. applied pyrolysis to materials derived from face masks and obtained pyrolysis oil with a high calorific value of 45.93 MJ/kg. Its chemical composition showed a hydrocarbon spectrum similar to gasoline. Such a highly calorific pyrolysis product can be considered suitable for energy recovery, for instance in cogeneration units for the production of heat and electricity. Similar calorific values were achieved by Li *et al*.^13^ during pyrolysis at temperatures between 456 and 466 °C. In this narrow temperature range, they achieved transformation of the main organic components of surgical masks while producing carbon-rich pyrolysis oil with a calorific value of 43.5 MJ/kg.

These findings corroborate the potential of pyrolysis as an efficient strategy for transforming waste into high-value raw materials. The present study investigates the pyrolytic conversion of disposable face masks into valuable solid, liquid, and gaseous products. The process was examined at target temperatures ranging from 350 to 550 °C, with particular attention paid to the composition of the feedstock and the physicochemical characteristics of the resulting outputs. This research lays the groundwork for optimizing pyrolysis as a method for generating value-added products, thereby contributing both to waste volume reduction and the development of alternative energy sources.

## Methods

### Input material

**Fig. 1:**
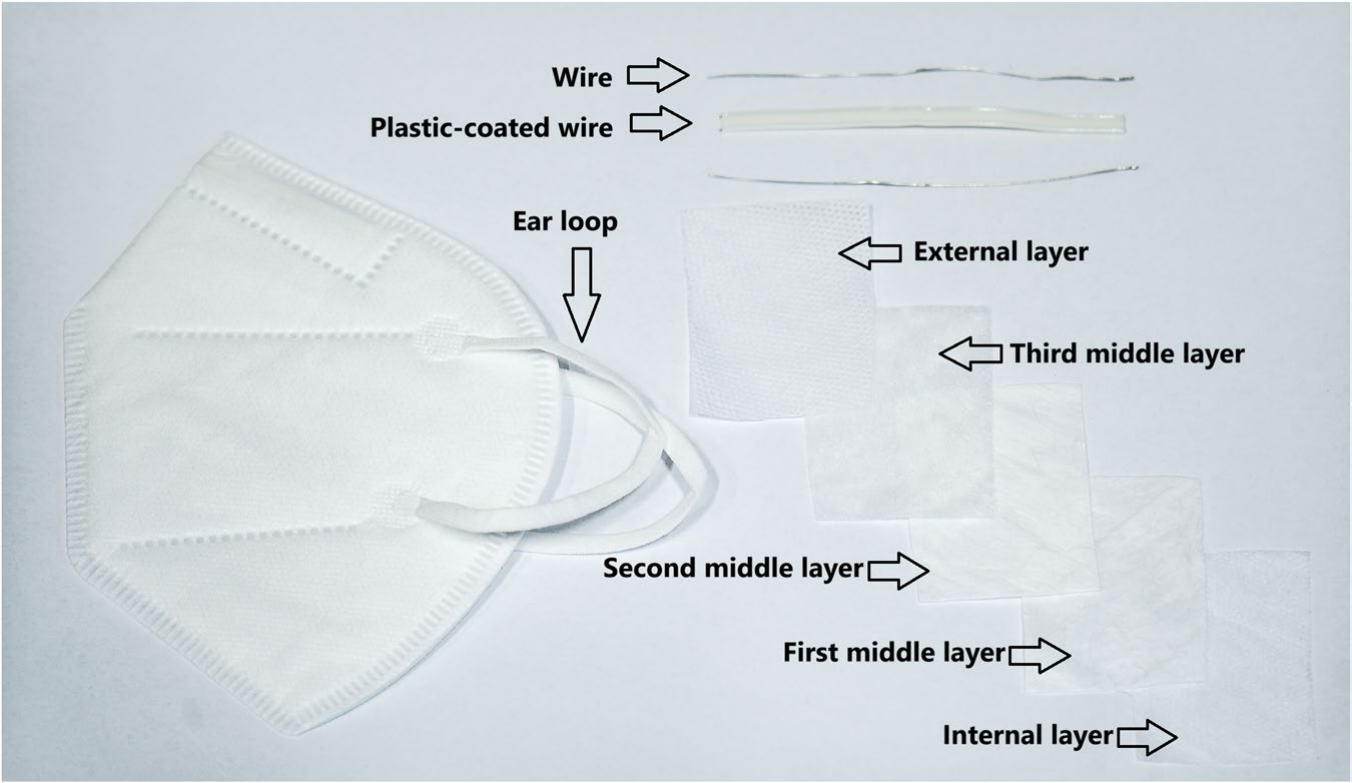
FFP2 face mask with specific part descriptions used in the pyrolysis process.

**Fig. 2:**
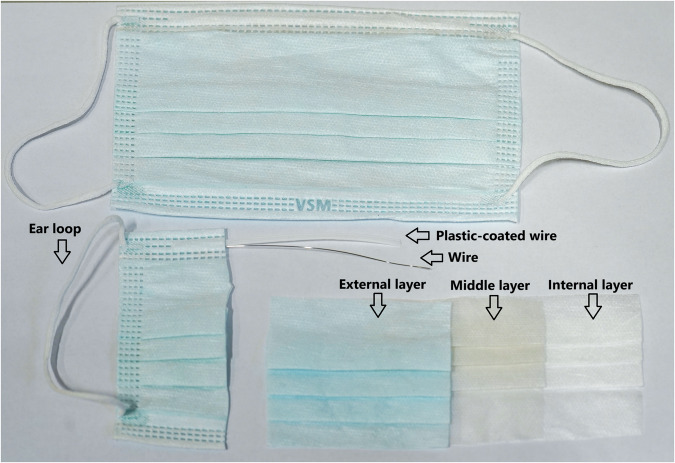
Ordinary face mask with specific part descriptions used in the pyrolysis process.

Used waste materials were these two types of disposable masks: FFP2 face masks (Fig. 1) and ordinary face masks (Fig. 2). The masks used in experiments were waste from production and were not contaminated with the virus due to health safety. Therefore, the decontamination process was not necessary. All components of the face masks were subjected to pyrolysis; no parts were removed prior to the process. All samples were subjected to 24 hours of natural air drying before pyrolysis treatment.

The elemental analysis of input materials was realized by LECO CHNS 628, thermogravimetric analysis by LECO TGA 701, calorific value by LECO AC 600 and FTIR by Thermo Scientific Nicolet iS10. The methodology of these devices is described in more detail in chapter 2.3. Data acquisition.

### Pyrolysis process

**Fig. 3:**
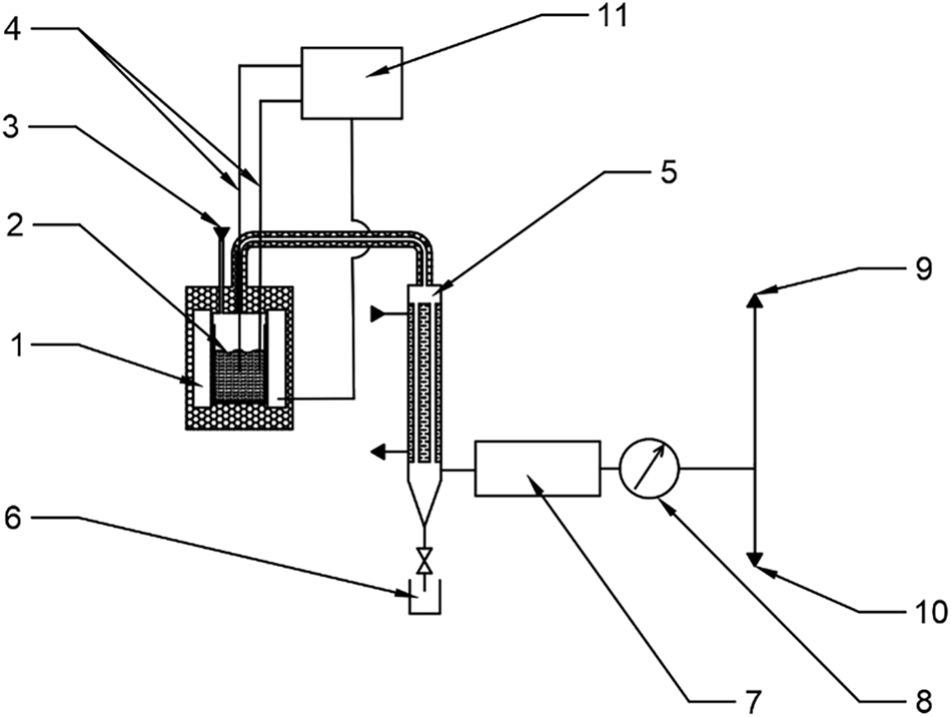
A schematic diagram of the experimental device: 1 Heater, 2 Sample, 3 Inert gas input, 4 Thermocouples, 5 Cooler, 6 Pyrolysis oil output, 7 Gas product purification, 8 Flow meter, 9 Gas analysis, 10 Gas output, 11 System controller.

Approximately 200 g of the sample was loaded into a batch pyrolysis unit. Subsequently, the reactor containing the sample was purged with nitrogen for 10 minutes. Afterward, the reactor heating was initiated to the various desired temperatures (350, 400, 450, 500 and 550 °C). Vapor condensation from the reactor was conducted using a tubular heat exchanger, and the liquid product was collected in the bottom of the condenser. The heating rate was set to 5 °C/min. The thermochemical process was monitored using two thermocouples placed within the raw material: one near the heated reactor wall and the other in the center of the reactor. The thermocouple near the wall served as the primary control sensor, and the temperature difference between the thermocouples did not exceed 5 °C throughout the process. The pyrolysis process was carried out at atmospheric pressure. The schematic diagram of the experimental device is shown in Fig. 3. The gaseous product was analyzed by Gas Chromatography/Flame Ionization Detector.

### Data acquisition

Quantitative elemental analysis of carbon (C), hydrogen (H), nitrogen (N), and sulfur (S) in the feedstock, solid residue (char), and liquid pyrolysis products were performed using a LECO CHNS 628 analyzer. This analyzer operates on the principle of combusting a defined amount of the sample, approximately 100 mg, in an oxygen atmosphere at a temperature of 950 °C. Combustion in pure oxygen leads to the decomposition of organic components and the formation of oxidation products, which are subsequently analyzed. Detection is carried out using non-dispersive infrared (NDIR) cells for CO_2_ and H_2_O, and a thermal conductivity (TC) cell for N_2_. The analyzers are calibrated using standard substances of known composition through calibration curve methods. For the incombustible residue remaining after elemental analysis, the content of inorganic elements was determined using an ARL^TM^ QUANT’X EDXRF spectrometer. The energy-dispersive X-ray fluorescence (EDXRF) analysis was carried out under vacuum conditions, with data evaluation performed using UniQuant software based on fundamental parameter algorithms. This method enabled the detection of elements across a wide range of atomic numbers.

Thermogravimetric analysis (TGA) was performed using a LECO TGA 701. A 1 g sample was heated at a rate of 5 °C/min under different atmospheric conditions to determine specific sample parameters. Initially, the sample was placed in an open alumina crucible and heated in a nitrogen (N_2_) atmosphere to 105 °C, where it was held until a constant weight was reached to determine the moisture content. The crucible was then covered with a lid, and the temperature was increased to 900 °C while maintaining a nitrogen atmosphere for 7 minutes to determine the volatile matter content. Subsequently, the sample was cooled to 550 °C, the lid was removed, and an oxidizing oxygen (O_2_) atmosphere was introduced. The temperature was then increased to 815 °C. The weight loss within this temperature range corresponded to the fixed carbon content, and the remaining mass was attributed to ash content. An exception is the analysis of pyrolysis oil, where the first two steps of TGA (heating in a nitrogen atmosphere to 105 °C and subsequently to 900 °C) yield a combined value for volatile matter content and water. This is because pyrolysis oil may contain components with boiling points below 105 °C, which evaporate together with water during heating. Therefore, the actual volatile matter content is determined indirectly—by subtracting the water content, which is measured separately using the standard automatic volumetric Karl Fischer titration method. TGA analysis was employed to determine the moisture content, ash content, volatile matter, and fixed carbon content for the input material, the char, and the oil after the pyrolysis process.

A LECO AC 600 semi-automatic calorimeter was used to determine the higher calorific value. Approximately 0.5 g samples were combusted in a controlled environment. The released heat is proportional to the calorific value of the substance. Liquid samples were combusted within a gelatin capsule. The higher calorific value measurements were realized for the input material, the char, and the oil after the pyrolysis process.

Sulphur content was verified by using an Eco IC ion chromatograph (Metrohm). The sample was weighed into a gelatine capsule and combusted in a calorimetric bomb. The combustion residue was then dissolved in hydrogen peroxide and diluted to a defined volume. The solution was subsequently analysed by ion chromatography.

Oils formed during pyrolysis experiments conducted at 500 °C on mask and respirator samples were analyzed using a gas chromatograph-mass spectrometer (GC-MS) from Agilent Technologies. The instrument was equipped with an Agilent 8890 GC and a 5977B MS detector. An HP-5 column was used to separate the analytes within the samples. The oils were dissolved in dichloromethane prior to analysis.

ATR (Attenuated Total Reflectance) was used for the analysis of the input sample. This technique is employed in infrared spectroscopy using a Thermo Nicolet iS10 Fourier Transform Infrared Spectrometer. Infrared radiation is directed into a high-refractive-index crystal (typically diamond), and the sample is pressed against its surface. The IR beam undergoes internal reflections within the crystal, generating an evanescent wave that penetrates a few micrometers into the sample, enabling surface-level spectral analysis. All parts of the FFP2 respirators and face masks shown in Figs. 1, 2 were analysed using this method.

The gaseous composition following the pyrolysis experiment was analysed using a gas chromatograph equipped with a flame ionization detector and a thermal conductivity detector (GC/FID/TCD), specifically a YL 6100 GC (Young Lin Bldg.). A ShinCarbon ST column (Micropacked) was employed for the separation of analytes within the gaseous samples.

## Data Records

The dataset comprising measurements of the technical and physicochemical properties of the feedstock, the pyrolysis process, and all resulting pyrolysis products is available under the title Thermal Degradation of Face Mask Waste through Pyrolysis at Different Temperatures for Value-Added Products^14^. The data are thematically divided into four.xlsx files, which are further categorized by topic. The distribution scheme is shown below.

1. FTIR analysis_FFP2_fm
  1.1. FTIR analysis all parts of FFP_fm
2. FTIR analysis_Ord_fm
  2.1 FTIR analysis all parts of Ord_fm
3. TGA analysis_FFP2_fm and Ord_fm
4. Physicochemical properties - raw material and pyrolysis products
  4.1. Physicochemical properties (PCP) of raw materials
  4.2. Mass balance of pyrolysis process
  4.3. Physicochemical properties (PCP) of pyrolysis char
  4.4. Physicochemical properties (PCP) of pyrolysis oil
  4.5. Physicochemical properties (PCP) of pyrolysis gas

## Technical Validation

All parts of the experimental research were conducted with the utmost care and using the best available methods for control and calibration. For the pyrolysis process, a statistically representative amount of FFP2 and ordinary face mask samples was selected. A detailed mass analysis of the input materials (FFP2_fm and Ord_fm) and their components was carried out using a Kern ABT 120-5DNM balance, featuring a maximum capacity of 120 g, readability of 0.1 mg, and linearity of ±0.2 mg. The mass balance of the pyrolysis process (solid residue and liquid product) was determined by a differential method using a KERN EWJ balance with a maximum capacity of 3000 g, readability of 10 mg, and linearity of ±50 mg. The amount of gas was measured using a newly certified membrane gas flow meter.

All analytical techniques were periodically serviced by manufacturer-accredited technicians in accordance with the device manufacturer’s recommendations. The LECO CHNS 628 elemental analyser is calibrated using phenylalanine (CAS 63-91-2) and ethylenediaminetetraacetic acid (EDTA, CAS 60-00-4) standards. For elemental analysis, each representative sample was measured in triplicate, and the average value was calculated from the results.

The calorimeter LECO AC 600, used to determine the higher heating value (HHV), is calibrated by combusting a defined quantity of benzoic acid (CAS 65-85-0) with a known combustion enthalpy. For the determination of HHV, each representative sample was measured in triplicate, and the average value was calculated from the results.

A gas chromatograph with mass spectrometric detection (GC-MS) was used for the analysis of unknown substances, applying a spectral comparison method against a reference database supplied by the instrument manufacturer. A gas chromatograph equipped with TCD (thermal conductivity detector) and FID (flame ionization detector) for gaseous samples was calibrated using certified calibration gases with precisely defined compositions.

## Usage Notes

We assume that data on the physicochemical properties of face masks and respirators, combined with data on pyrolysis products at various temperatures, can, through advanced statistical analysis, lead to identifying optimal conditions for the disposal of face masks. By understanding the dependent variables and their equations, it will be possible to define the properties and quantities of individual products based on temperature and material properties, ensuring their potential applications. Face masks and respirators also contain varying amounts of additives to achieve the desired product properties (e.g., colour or flexibility). Analysis of the measured data may potentially identify which parts of face masks predominantly contain these additives and how they influence the products depending on the pyrolysis temperature.

## Data Availability

No code was used in this study.
